# The Tumor Microenvironment Score and Its Subsite-Specific Relevance in Colon Cancer

**DOI:** 10.1097/PAS.0000000000002599

**Published:** 2026-08-25

**Authors:** Mari J. Jawad, Vilja V. Tapiainen, Ville K. Äijälä, Päivi H. Sirniö, Anne E. Tuomisto, Hanna Elomaa, Maarit Ahtiainen, Olli Helminen, Erkki-Ville Wirta, Toni T. Seppälä, Jan Böhm, Jukka-Pekka Mecklin, Henna Karjalainen, Meeri Kastinen, Timo Väisänen, Vesa-Matti J. Pohjanen, Markus J. Mäkinen, Jukka M. Rintala, Heikki Huhta, Juha P. Väyrynen

**Affiliations:** * Translational Medicine Research Unit, Medical Research Center Oulu, Oulu University Hospital, and University of Oulu; §§ Department of Pathology; ∥∥ Department of Surgery, Oulu University Hospital, Oulu; † Research Program in Systems Oncology; ¶ Department of Gastrointestinal Surgery, Helsinki University Central Hospital; # Applied Tumour Genomics, Research Program Unit, University of Helsinki, Helsinki; ‡ Central Finland Biobank, Hospital Nova of Central Finland; ** Department of Pathology, Hospital Nova of Central Finland; †† Department of Education and Research, Well Being Services County of Central Finland; ‡‡ Faculty of Sport and Health Sciences, University of Jyväskylä, Jyväskylä; § Department of Gastroenterology and Alimentary Tract Surgery; ∥ Center for Cancer Eradication Research, Faculty of Medicine and Health Technology, Tampere University and Tays Cancer Centre, Tampere University Hospital, Tampere, Finland

**Keywords:** tumor microenvironment, right-sided colon cancer, left-sided colon cancer, histopathology

## Abstract

Colon cancer is a major global malignancy with significant mortality. Right-sided (RCC) and left-sided colon cancers (LCC) exhibit distinct clinicopathological characteristics. Tumor microenvironment score (TMS) combines Klintrup-Mäkinen grade, tumor stroma percentage, and tumor budding, classifying cases into 4 groups (TMS 0-3). Evaluation of TMS is an integrative method for prognostic classification; however, further validation is needed, and differences between RCC and LCC remain unclear. A discovery cohort (n=940) and a validation cohort (n=613) were retrospectively included. TMS was evaluated from hematoxylin-eosin-stained slides. Five-year disease-specific survival (DSS) was analyzed using life-table methods and Cox regression. Associations with clinicopathological variables were assessed. In both the discovery and validation cohorts, higher TMS was associated with worse 5-year DSS in univariable analyses for both RCC and LCC. In multivariable analysis, high TMS (TMS3 vs. TMS0) remained independently associated with survival in the discovery cohort (RCC—HR: 2.47, 95% CI: 1.36-4.46, *P*=0.003; LCC—HR: 3.08, 95% CI: 1.46-6.45, *P*=0.003). In the validation cohort, the prognostic impact of TMS was more pronounced in RCC (HR: 4.35, 95% CI: 2.32-8.14, *P*<0.001) than in LCC (HR: 2.21, 95% CI: 0.81-6.03, *P*=0.12). TMS also appeared to provide improved prognostic stratification compared with the Glasgow microenvironment score (combination of Klintrup-Mäkinen grade and tumor stroma percentage). In conclusion, TMS serves as an integrative histopathological marker with potential to improve prognostic stratification in colon cancer, particularly in right-sided tumors.

Colon cancer is the fourth most diagnosed cancer worldwide and the fifth leading cause of cancer-related death.^1^ As precision oncology advances, incorporating tumor pathology and genetics can improve prognostication and guide treatment beyond traditional TNM staging.^2^ Additional tumor-derived biomarkers, including mismatch-repair (MMR) status^3^ and the Immunoscore,^4^ are increasingly recognized. Colorectal cancer (CRC) has also been stratified into 4 molecular subtypes according to gene-expression profiles (consensus molecular subtypes CMS1-4), which may carry prognostic significance for overall survival in metastatic disease.^5^ However, many of these prognostic evaluations rely on complex transcriptional and mutational profiling or multiantibody immunostaining, which remain challenging to implement in routine diagnostics.^6,7^

Histological evaluation remains the foundation of pathological staging and subsequent clinical decision-making.^3^ Therefore, prognostic markers evaluated from routine hematoxylin-eosin (H&E)-stained sections offer a practical approach for clinical use.^6^ The Klintrup-Mäkinen grade (KMG),^8^ tumor stroma percentage (TSP),^9^ and tumor budding (TB) are among the most-studied H&E-based prognostic markers;^10^ of these, only TB is currently used in routine clinical practice. The Glasgow Microenvironment Score (GMS) integrates the inflammatory cell response (KMG) with tumor stroma (TSP) assessment and has been shown to provide improved risk stratification within a heterogeneous cohort of CRC patients.^11^ The tumor microenvironment score (TMS) combines KMG, TSP, and TB and has been shown to serve as a more effective prognostic marker than GMS alone.^6^

Colon cancers are commonly classified as right-sided (RCC) or left-sided (LCC) relative to the splenic flexure. RCC and LCC differ in molecular characteristics and clinical outcomes, and RCC is associated with poorer overall survival in metastatic disease.^12^ Tumor sidedness, together with molecular profiling, guides treatment selection, including anti-EGFR monoclonal antibodies as conversion therapy for RAS wild-type metastatic LCC when complete resection is intended.^13^ MMR/microsatellite instability (MSI) status guides treatment selection across stages,^14^ and deficient (dMMR) or MSI-high (MSI-H) tumors are predominantly right-sided.^15^

The prognostic significance of TMS has not been separately characterized for RCC and LCC. Therefore, the aim of this study was to validate the application of TMS in colon cancer, with emphasis on subsite-specific relevance (RCC vs. LCC) within the colon, and to compare its prognostic performance with GMS.

## MATERIALS AND METHODS

The study was performed in accordance with the Declaration of Helsinki. For the discovery cohort, the study was conducted under approval from the Regional Medical Research Ethics Committee of the Wellbeing Services County of Central Finland (13U/2011, 1/2016, 8/2020, 2/2023), Central Finland Biobank (BB23-0172), and Fimea (FIMEA/2023/ 001573), and for the validation cohort, under approval from the Wellbeing Services County of North Ostrobothnia (EETTMK 81/2008) and Valvira (Dnro 10832/06.01.03.01/2014).

### Patient Cohorts

A retrospective discovery cohort consisted of surgically treated colon cancer patients from Central Finland Central Hospital (CFCH) (2000 to 2015, n=940). The validation cohort comprised patients from Oulu University Hospital (OUH) (2006 to 2015, n=450) and Lapland Central Hospital (LCH) (2005 to 2015, n=167). Four cases were excluded due to a lack of a sufficiently evaluable invasive margin for TMS and GMS assessment, resulting in a final validation cohort of 613 patients. Rectal cancers were excluded. Baseline characteristics of the cohorts are shown in Table 1.

**TABLE 1:** Baseline Characteristics of the Discovery and Validation Cohorts in Relation to Tumor Sidedness

	Discovery cohort			Validation cohort		
	RCC (n=536), N (%)	LCC (n=404), N (%)	*P*	RCC (n=361), N (%)	LCC (n=252), N (%)	*P*
Age, median (IQR)	74 (65-81)	71 (62-78)	<0.001	75 (66-82)	72 (63-79)	<0.001
Sex, female	290 (54.1)	192 (47.5)	0.046	205 (56.8)	117 (46.4)	0.012
ASA			0.019			0.45
1	18 (3.4)	21 (5.2)		14 (4.0)	14 (5.6)	
2	187 (34.9)	174 (43.1)		103 (29.2)	81 (32.5)	
3	254 (47.4)	162 (40.1)		189 (53.5)	118 (47.4)	
4	77 (14.4)	47 (11.6)		47 (13.3)	36 (14.5)	
NA				8	3	
T-class			0.41			0.25
1	20 (3.7)	22 (5.4)		10 (2.8)	12 (4.8)	
2	80 (14.9)	49 (12.1)		83 (23.0)	45 (17.9)	
3	334 (63.6)	255 (63.1)		199 (55.1)	150 (59.5)	
4	102 (19.0)	78 (19.3)		69 (19.1)	45 (17.9)	
N-class			0.017			0.33
0	296 (55.2)	230 (56.9)		211 (58.4)	146 (57.9)	
1	127 (23.7)	116 (28.7)		88 (24.4)	73 (29.0)	
2	113 (21.1)	58 (14.4)		59 (16.3)	30 (11.9)	
Nx				3 (0.8)	3 (1.2)	
M-class			0.42			0.18
0	465 (86.8)	343 (84.9)		304 (84.2)	198 (78.6)	
1	71 (13.2)	61 (15.1)		55 (15.2)	53 (21.0)	
Mx				2 (0.6)	1 (0.4)	
Stage			0.79			0.24
1	80 (14.9)	56 (13.9)		77 (21.3)	47 (18.7)	
2	204 (38.1)	158 (39.1)		125 (34.6)	89 (35.3)	
3	181 (33.8)	129 (31.9)		104 (28.8)	63 (25.0)	
4	71 (13.2)	61 (15.1)		55 (15.2)	53 (21.0)	
Grade			<0.001			<0.001
Low	389 (72.6)	367 (90.8)		309 (85.6)	237 (94.0)	
High	147 (27.4)	37 (9.2)		52 (14.4)	15 (6.0)	
Histological subtype			<0.001			<0.006
Adenocarcinoma	464 (86.6)	381 (94.6)		323 (89.5)	242 (96.0)	
Mucinous carcinoma	51 (9.5)	19 (4.7)		32 (8.9)	10 (4.0)	
Signet ring cell carcinoma	21 (3.9)	4 (1.0)		6 (1.7)	0	
Lymphatic or vascular invasion, yes	119 (22.2)	86 (21.3)	0.74	101 (28.0)	61 (24.2)	0.30
MMR status			<0.001			<0.001
Proficient	390 (72.8)	384 (95.0)		249 (69.0)	238 (94.4)	
Deficient	146 (27.2)	20 (5.0)		112 (31.0)	14 (5.6)	
*BRAF* mutation, yes	159 (29.8)	20 (5.0)	<0.001	99 (27.4)	11 (4.4)	<0.001
N.A.	2					
Tumor budding			0.11			<0.001
Low (0-4)	356 (66.4)	268 (66.3)		219 (60.7)	155 (61.5)	
Intermediate (5-9)	101 (18.8)	92 (22.8)		65 (18.0)	76 (30.2)	
High (10 or more)	79 (14.7)	44 (10.9)		77 (21.2)	21 (8.3)	
KMG			0.19			0.039
Low (0-1)	305 (56.9)	247 (61.1)		199 (55.1)	160 (63.5)	
High (2-3)	231 (43.1)	157 (38.9)		162 (44.9)	92 (36.5)	
TSP			0.33			0.44
Low (≤50%)	365 (68.1)	263 (65.1)		182 (50.4)	135 (53.6)	
High (>50%)	171 (31.9)	141 (34.9)		179 (49.6)	117 (46.4)	

ASA indicates American Society of Anesthesiologists Physical Status Classification System, KMG, the Klintrup-Mäkinen grade, LCC, left-sided colon cancer, MMR, mismatch-repair, NA, not available, RCC, right-sided colon cancer, TSP, tumor stroma percentage.

### Histopathological Scoring

Scoring of the KMG, TSP, and TB in the CFCH cohort was performed by J.P.V. and V.K.Ä. In the OUH cohort, scoring was performed by M.J.J., V.V.T., and V.K.Ä., and in the LCH cohort by M.J.J. The original diagnostic H&E slides were retrieved from the pathology archives of the participating hospitals and were scanned using Aperio AT2 (Leica Biosystems, Wetzlar, Germany) and Hamamatsu NanoZoomer S60/NanoZoomer-XR (Hamamatsu Photonics K.K., Hamamatsu, Japan). H&E-stained slides from the deepest invasive front of the tumor were assessed. The KMG, TSP, and TB were evaluated as previously described;^8–10^ see Figure 1 for an illustration of the categorization. The KMG categorizes tumors into 4 groups based on the overall inflammatory reaction at the invasive margin (0=absent, 1=weak, 2=moderate, 3=severe). Scores were further grouped into 2 categories: 0 to 1=mild and 2 to 3=high-grade inflammation.^8^ Tumor budding was evaluated as the number of single tumor cells or 2- to 4-cell clusters in a 0.785 mm^2^ hotspot^10^ and categorized as low (0 to 9 buds) and high (≥10 buds). TSP was assessed as the proportion of stromal area within a 2 mm-diameter hotspot, with ≤50% classified as stroma-low and >50% as stroma-high.^9^ Evaluations were performed blinded to study outcomes.

**FIGURE 1:**
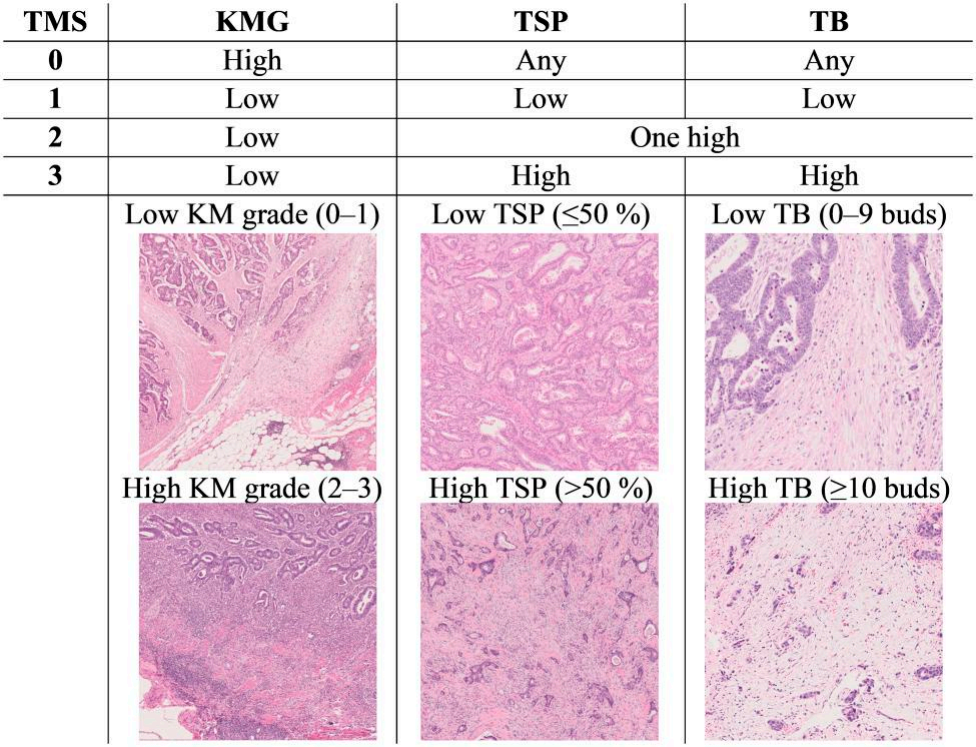
TMS classification based on Klintrup-Mäkinen grade (KMG), tumor stroma percentage (TSP), and tumor budding (TB), with representative areas of hematoxylin-eosin-stained sections.

### Tumor Microenvironment Score

TMS was classified as follows: TMS0, high KMG (KMG 2-3); TMS1, low KMG (KMG 0-1) with low TSP (≤50%) and low TB (0-9 buds); TMS2, low KMG with either high TSP (>50%) or high TB (≥10 buds), but not both; and TMS3, low KMG with both high TSP and high TB.^6^

### Glasgow Microenvironment Score

GMS was classified as follows: GMS0, high KMG (KMG 2-3); GMS1, low KMG (KMG 0-1) with low TSP (≤50%); and GMS2, low KMG with high TSP (>50%).^11^

### Outcomes

The primary outcome was the association between TMS and 5-year disease-specific survival (DSS), defined as the time from the date of surgery to colon cancer-related death, analyzed separately for RCC and LCC. Secondary outcomes were the associations between TMS and clinicopathological characteristics. Primary analyses were also performed for GMS.

### Statistical Analysis

The χ^2^ test was used to compare categorical variables, and the *t* test to compare continuous variables. DSS was estimated using life-table and Kaplan-Meier methods, and groups were compared using the log-rank test. Cox proportional hazards regression was used to estimate hazard ratios (HRs) with 95% CIs. Multivariable models were adjusted for *BRAF* status (wild-type/mutated), stage (I to IV), MMR status (proficient/deficient), age (continuous), sex (male/female), ASA class (1 to 4), and lymphatic and/or vascular invasion (no/yes). *P*<0.05 was considered statistically significant. Missing ASA data in the discovery cohort (n=76) were addressed using multiple imputation (20 imputations). For all other variables, the extent of missing data was negligible (Table 1), and no imputation was performed. For subgroup analyses stratified by pMMR/dMMR status, ASA class was dichotomized into ASA I to II and ASA III to IV, and tumor stage was grouped as stage I to II versus stage III to IV. In subgroup analyses including stage I to IV patients, the ASA classification was dichotomized accordingly. IBM SPSS Statistics 27.0 (IBM Corp., Armonk, NY) was used for statistical analysis.

## RESULTS

### Primary Outcomes

#### Tumor Microenvironment Score

In the discovery cohort, 388 (41%) cases were classified as TMS0, 250 (27%) as TMS1, 224 (24%) as TMS2, and 78 (8%) as TMS3. In the validation cohort, the corresponding values were 254 (41%) for TMS0, 160 (26%) for TMS1, 143 (23%) for TMS2, and 56 (9%) for TMS3.

In univariable analyses, higher TMS categories were associated with worse DSS in both RCC and LCC and in both the discovery and validation cohorts (Table 2). In Kaplan-Meier analyses, 5-year DSS in the discovery cohort for RCC was 88%, 65%, 55%, and 36% (*P*<0.001; Fig. 2A) and for LCC 89%, 76%, 53%, and 38% (*P*<0.001; Fig. 2B) for TMS 0-3, respectively. In the validation cohort, the corresponding 5-year DSS estimates were 85%, 65%, 59%, and 27% for RCC (*P*<0.001; Fig. 2C) and 85%, 74%, 41%, and 57% for LCC (*P*<0.001; Fig. 2D).

**TABLE 2:** Univariable and Multivariable Analysis of 5-Year Disease-Specific Survival According to the Tumor Microenvironment Score (TMS) in Right-Sided Colon Cancer (RCC) and Left-Sided Colon Cancer (LCC) in the Discovery and Validation Cohort

	Univariable analysis (RCC)		Multivariable analysis (RCC)		Univariable analysis (LCC)		Multivariable analysis (LCC)	
	HR	*P*	HR	*P*	HR	*P*	HR	*P*
Discovery cohort								
TMS (ref TMS0)		<0.001		0.007		<0.001		0.004
TMS1	3.47 (2.13-5.65)	<0.001	2.21 (1.34-3.66)	0.002	2.35 (1.26-4.44)	0.007	1.65 (0.86-3.18)	0.13
TMS2	4.62 (2.87-7.43)	<0.001	2.14 (1.28-3.59)	0.004	5.69 (3.21-10.08)	<0.001	2.77 (1.49-5.15)	0.001
TMS3	7.78 (4.58-13.24)	<0.001	2.47 (1.36-4.46)	0.003	8.95 (4.56-17.57)	<0.001	3.08 (1.46-6.45)	0.003
Validation cohort								
TMS (ref TMS0)		<0.001		<0.001		<0.001		0.15
TMS1	2.70 (1.55-4.70)	<0.001	1.91 (1.06-3.45)	0.031	1.94 (0.97-3.90)	0.062	1.53 (0.74-3.17)	0.25
TMS2	3.05 (1.77-5.25)	<0.001	1.93 (1.06-3.51)	0.030	5.43 (2.91-10.27)	<0.001	2.16 (1.10-4.26)	0.026
TMS3	8.22 (4.74-14.27)	<0.001	4.35 (2.32-8.14)	<0.001	3.29 (1.25-8.65)	0.016	2.21 (0.81-6.03)	0.12

**FIGURE 2:**
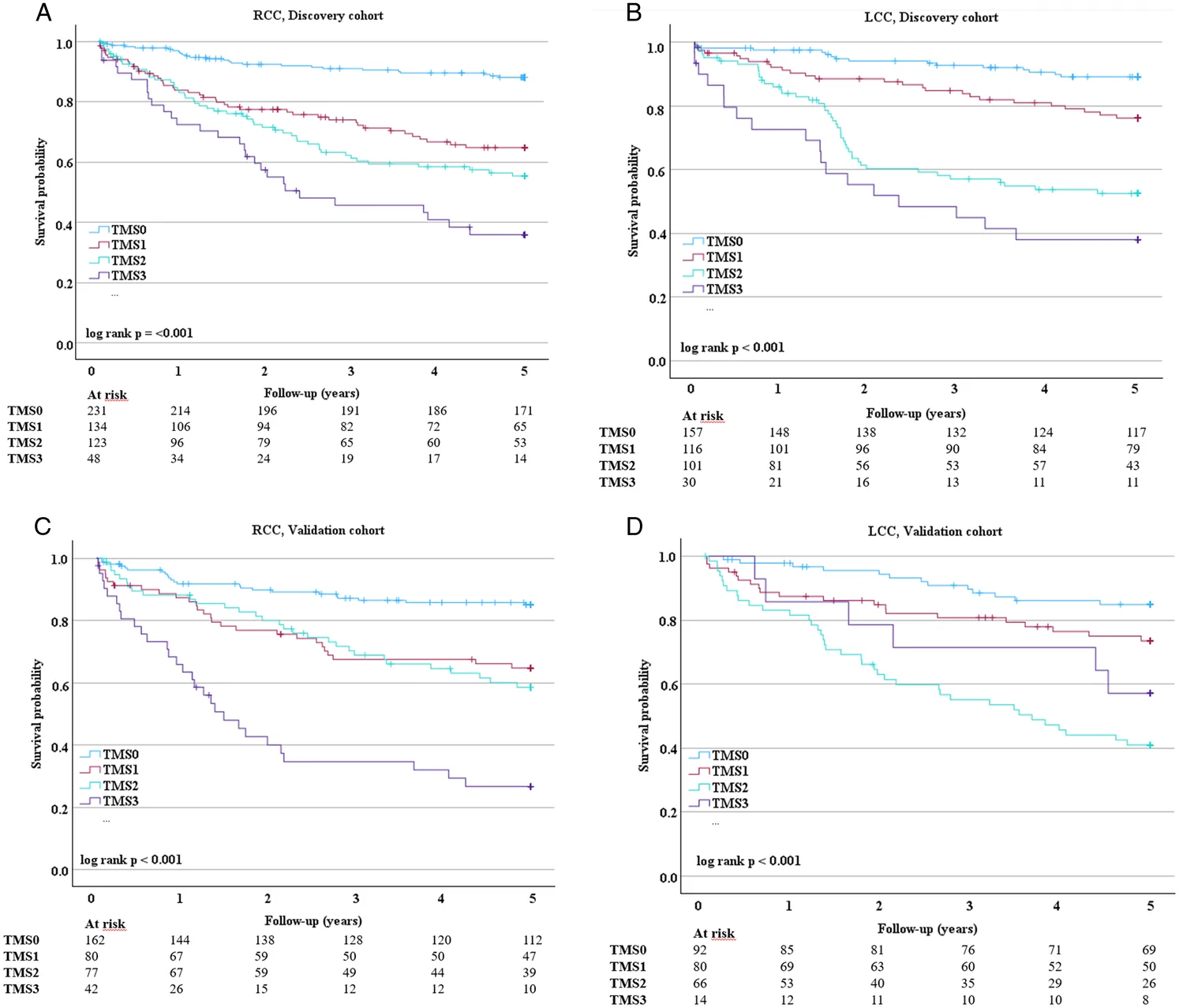
The association between the tumor microenvironment score (TMS) and disease-specific survival in (A) right-sided colon cancer (RCC) in the discovery cohort, (B) left-sided colon cancer (LCC) in the discovery cohort, (C) RCC in the validation cohort, and (D) LCC in the validation cohort.

In multivariable analyses, TMS remained independently associated with DSS in RCC in both cohorts (Table 2). The HR for TMS3 versus TMS0 was 2.47 (95% CI: 1.36-4.46, *P*=0.003) in the discovery cohort and 4.35 (95% CI: 2.32-8.14, *P*<0.001) in the validation cohort. For LCC, TMS remained independently associated with DSS in the discovery cohort but not in the validation cohort (Table 2). The HRs for TMS3 versus TMS0 were 3.08 (95% CI: 1.46-6.45, *P*=0.003) and 2.21 (95% CI: 0.81-6.03, *P*=0.12), respectively. Complete multivariable results are provided in Supplementary Table S1 (Supplemental Digital Content 1, https://links.lww.com/PAS/C395).

#### Glasgow Microenvironment Score

In the discovery cohort, 388 (41%) cases were classified as GMS0, 287 (31%) as GMS1, and 265 (28%) as GMS2. In the validation cohort, the corresponding values were 254 (41%), 166 (27%), and 193 (31%), respectively.

In univariable analyses, a higher GMS category was associated with worse DSS in both RCC and LCC in both cohorts (Table 3). In Kaplan-Meier analyses, 5-year DSS in the discovery cohort for RCC was 88%, 63%, and 49% (*P*<0.001; Fig. 3A), and for LCC was 89%, 73%, and 50% (*P*<0.001; Fig. 3B) for GMS0-2, respectively. In the validation cohort, the corresponding 5-year DSS estimates were 85%, 65% and 47% for RCC (*P*<0.001; Fig. 3C) and 85%, 73% and 44% for LCC (*P*<0.001; Fig. 3D).

**TABLE 3:** Univariable and Multivariable Analysis of 5-Year Disease-Specific Survival According to the Glasgow Microenvironment Score (GMS) in Right-Sided Colon Cancer (RCC) and Left-Sided Colon Cancer (LCC) in the Discovery and Validation Cohort

	Univariable analysis (RCC)		Multivariable analysis (RCC)		Univariable analysis (LCC)		Multivariable analysis (LCC)	
	HR	*P*	HR	*P*	HR	*P*	HR	*P*
Discovery cohort								
GMS (ref GMS0)		<0.001		0.002		<0.001		0.003
GMS1	3.69 (2.30-5.89)	<0.001	2.35 (1.45-3.82)	<0.001	2.75 (1.51-5.00)	<0.001	1.78 (0.95-3.35)	0.073
GMS2	5.48 (3.49-8.61)	<0.001	2.07 (1.25-3.43)	0.005	6.20 (3.55-10.82)	<0.001	2.80 (1.52-5.14)	<0.001
Validation cohort								
GMS (ref GMS0)		<0.001		0.003		<0.001		0.062
GMS1	2.73 (1.58-4.72)	<0.001	1.98 (1.11-3.53)	0.021	2.03 (1.01-4.05)	0.045	1.53 (0.75-3.14)	0.25
GMS2	4.43 (2.73-7.20)	<0.001	2.59 (1.51-4.45)	<0.001	4.93 (2.65-9.18)	<0.001	2.18 (1.13-4.24)	0.021

**FIGURE 3:**
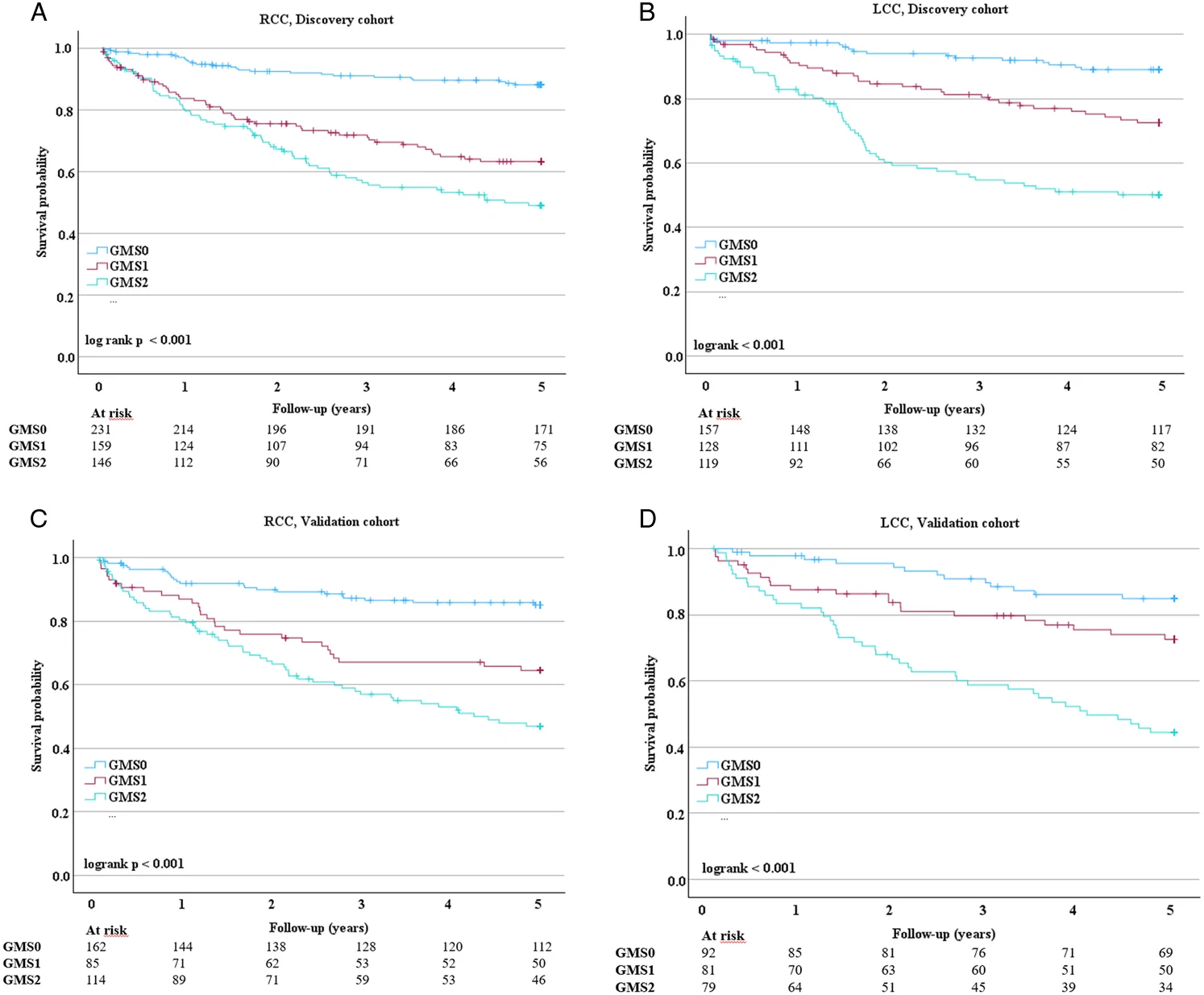
The association between the Glasgow microenvironment score (GMS) and disease-specific survival in (A) right-sided colon cancer (RCC) in the discovery cohort, (B) left-sided colon cancer (LCC) in the discovery cohort, (C) RCC in the validation cohort, and (D) LCC in the validation cohort.

In multivariable analyses, GMS2 remained independently associated with worse DSS in RCC and LCC in both cohorts (Table 3). However, for LCC, the adjusted association for GMS1 (vs. GMS0) was not statistically significant in either the discovery cohort (HR: 1.78, 95% CI: 0.95-3.35, *P*=0.073) or the validation cohort (HR: 1.53, 95% CI: 0.75-3.14, *P*=0.25) (Table 3). Complete multivariable results are provided in Supplementary Table S2 (Supplemental Digital Content 1, https://links.lww.com/PAS/C396).

Compared with GMS, higher TMS categories identified subgroups with poorer prognosis. In RCC, 5-year DSS for TMS3 was 36% in the discovery cohort and 27% in the validation cohort, whereas the GMS2 estimates were 49% and 47%, respectively. Similarly, in LCC, 5-year DSS for TMS3 was 38% and 57%, while the corresponding GMS2 estimates were 50% and 44% in the discovery and validation cohorts, respectively.

#### Secondary Outcomes

In the discovery cohort, TMS, TB, KMG, and TSP were not significantly associated with tumor sidedness (TMS *P*=0.360; TB *P*=0.114; KMG *P*=0.192; TSP *P*=0.334) (Tables 1 and 4). In the validation cohort, TB and KMG were associated with sidedness, with RCC exhibiting higher TB (*P*<0.001) and LCC showing weaker KMG (*P*=0.039) (Table 1). In the validation cohort, TMS0 and TMS3 were more frequently observed in RCC (*P*=0.002) (Table 4).

**TABLE 4:** The Association Between the Tumor Microenvironment Score (TMS) and Clinicopathological Characteristics in the Discovery and Validation Cohort

Discovery cohort	TMS0 (n=388), N (%)	TMS1 (n=250), N (%)	TMS2 (n=224), N (%)	TMS3 (n=78), N (%)	*P*
Sex, female	188 (48.5)	124 (49.6)	112 (50.0)	34 (43.6)	0.79
Tumor subsite					0.36
RCC	231 (59.5)	134 (53.6)	123 (54.9)	48 (61.5)	
LCC	157 (40.5)	116 (46.4)	101 (45.1)	30 (38.5)	
T-class					
T1-2	127 (32.7)	35 (14.0)	7 (3.1)	2 (2.6)	<0.001
T3-4	261 (67.3)	215 (86.0)	217 (96.9)	76 (97.4)	
N-class					<0.001
N0	291 (75.0)	138 (55.2)	78 (34.8)	19 (24.4)	
N1	97 (25.0)	112 (44.8)	146 (65.2)	59 (75.6)	
M-class					<0.001
M0	376 (96.9)	211 (84.4)	171 (76.3)	50 (64.1)	
M1	12 (3.1)	39 (15.6)	53 (23.7)	28 (35.9)	
Stage					<0.001
1	103 (26.5)	27 (10.8)	4 (1.8)	2 (2.6)	
2	182 (46.9)	99 (39.6)	67 (29.9)	14 (17.9)	
3	91 (23.5)	85 (34.0)	100 (44.6)	34 (43.6)	
4	12 (3.1)	39 (15.6)	53 (23.7)	28 (35.9)	
MMR, deficient	91 (23.5)	44 (17.6)	27 (12.1)	4 (5.1)	<0.001
*BRAF* mutation, yes	79 (20.4)	51 (20.4)	33 (14.8)	16 (20.5)	0.32
NA	1		1		
Lymphatic or vascular invasion, yes	35 (9.0)	49 (19.5)	79 (35.3)	42 (53.8)	<0.001
Validation cohort	TMS0 (n=254), N (%)	TMS1 (n=160), N (%)	TMS2 (n=143), N (%)	TMS3 (n=56), N (%)	*P*
Sex, female	140 (55.1)	78 (48.8)	72 (50.3)	32 (57.1)	0.50
Tumor subsite					0.002
RCC	162 (63.8)	80 (50.0)	77 (53.8)	42 (75.0)	
LCC	92 (36.2)	80 (50.0)	66 (46.2)	14 (25.0)	
T-class					<0.001
T1-2	84 (33.1)	43 (26.9)	20 (14.0)	3 (5.4)	
T3-4	170 (66.9)	117 (73.1)	123 (86.0)	53 (94.6)	
N-class					<0.001
N0	171 (67.3)	104 (65.0)	71 (49.7)	12 (21.4)	
N1	83 (32.7)	56 (35.0)	72 (50.3)	44 (78.6)	
M-class					<0.001
M0	234 (92.1)	131 (81.9)	103 (72.0)	37 (66.1)	
M1	20 (7.9)	29 (18.1)	40 (28.0)	19 (33.9)	
Stage					<0.001
1	72 (28.3)	37 (23.1)	15 (10.5)	0	
2	94 (37.0)	61 (38.1)	48 (33.6)	11 (19.6)	
3	68 (26.8)	33 (20.6)	41 (28.7)	26 (46.4)	
4	20 (7.9)	29 (18.1)	39 (27.3)	19 (33.9)	
MMR, deficient	81 (31.9)	25 (15.6)	13 (9.1)	7 (12.5)	<0.001
*BRAF* mutation, yes	53 (20.9)	27 (16.9)	18 (12.6)	12 (21.4)	0.18
Lymphatic or vascular invasion, yes	54 (21.3)	42 (26.3)	41 (28.7)	25 (44.6)	0.004

In both cohorts, higher TMS categories were associated with lymphatic or vascular invasion (discovery *P*<0.001; validation *P*=0.004), higher TNM classification (both *P*<0.001), and more advanced stage (both *P*<0.001) (Table 4). In both cohorts, MMR deficiency was associated with lower TMS categories (both *P*<0.001) (Table 4).

#### Subgroup Analyses Stratified by MMR Status and Tumor Stage

In subgroup analyses of pMMR patients, TMS demonstrated a stronger association with prognosis in RCC than in LCC. In multivariable analysis for RCC, the HR for TMS3 versus TMS0 was 2.94 (95% CI: 1.49-5.82, *P*=0.002) in the discovery cohort and 5.21 (95% CI: 2.58-10.53, *P*<0.001) in the validation cohort. For LCC, the corresponding multivariable HRs for TMS3 versus TMS0 were 5.13 (95% CI: 2.54-10.37, *P*<0.001) in the discovery cohort and 1.89 (95% CI: 0.71-5.04, *P*=0.20) in the validation cohort. In addition, in LCC, the comparison of TMS1 versus TMS0 was not statistically significant in either the discovery cohort (HR: 1.59, 95% CI: 0.84-3.02, *P*=0.16) or the validation cohort (HR: 2.01, 95% CI: 0.98-4.11, *P*=0.055). (Table S3, Supplemental Digital Content 1, https://links.lww.com/PAS/C397).

In RCC patients with dMMR, the comparison of TMS3 versus TMS0 reached statistical significance only in univariable analysis in the validation cohort (HR: 5.75, 95% CI: 1.76-18.83, *P*=0.004), whereas statistical significance was not achieved in multivariable analysis (Table S3, Supplemental Digital Content 1, https://links.lww.com/PAS/C397). For LCC, Cox regression analysis was not feasible due to the small subgroup sizes (n=20 in the discovery cohort and n=14 in the validation cohort).

In stage I and IV patients, TMS did not demonstrate prognostic significance in either RCC or LCC. Among stage II RCC patients, multivariable analysis showed that the HR for TMS2 versus TMS0 was 5.20 (95% CI: 1.97-13.76, *P*<0.001) in the discovery cohort, while the HR for TMS3 versus TMS0 was 12.44 (95% CI: 2.07-74.86, *P*=0.006) in the validation cohort. In stage II LCC patients, statistical significance was not achieved in the discovery cohort, and Cox regression analysis was not feasible in the validation cohort due to small subgroup sizes. (Table S4, Supplemental Digital Content 1, https://links.lww.com/PAS/C398).

In stage III RCC patients, multivariable analysis showed that the HR for TMS3 versus TMS0 was 2.83 (95% CI: 1.16-6.93, *P*=0.023) in the discovery cohort and 3.90 (95% CI: 1.54-9.88, *P*=0.004) in the validation cohort. For stage III LCC patients, statistical significance was not achieved in the discovery cohort. In the validation cohort, multivariable analysis showed that the HR for TMS2 versus TMS0 was 21.75 (95% CI: 4.3-110.00, *P*<0.001). (Table S4, Supplemental Digital Content 1, https://links.lww.com/PAS/C398).

## DISCUSSION

In this study, we evaluated TMS separately for right- and left-sided colon cancer and showed that its prognostic impact was more pronounced in RCC than in LCC. Higher TMS categories were associated with poorer prognosis in both subsites, and TMS3 generally identified patients with the lowest 5-year DSS. In multivariable analyses, TMS remained independently associated with DSS in RCC in both the discovery and validation cohorts, whereas in LCC the association was less consistent and did not remain independently significant in the validation cohort. Compared with GMS, TMS appeared to provide additional prognostic stratification, particularly by identifying high-risk subgroups among patients with RCC. In subgroup analyses of pMMR patients, TMS demonstrated a stronger association with prognosis in RCC than in LCC. Also, in stages II and III, the prognostic impact of TMS appeared more pronounced in RCC than in LCC, although this was not consistent across all TMS categories.

Survival varies substantially within the same TNM stage, particularly in stages II and III. As precision oncology advances, integrating detailed pathological and genetic tumor features into clinical decision-making may enable more accurate prognostication and treatment strategies beyond conventional TNM-based classification.^2^ In this context, TMS may provide a structured histopathological tool for identifying patients with poorer prognosis, particularly in RCC.

The divergent immune microenvironments and signaling pathways of right- and left-sided CRC emphasize the need for tailored therapeutic strategies.^16^ However, the conventional right versus left classification has been questioned, as it may not fully capture regional biological heterogeneity along the colon. The rectosigmoid region appears distinct, while the transverse colon may share features with left-sided tumors, and some descending colon characteristics resemble right-sided tumors.^12^ CMS2 and CMS4 are more prevalent in left-sided CRC, whereas CMS1 is more common in right-sided CRC. Moreover, molecular changes may occur along a continuum rather than forming a strict division between left- and right-sided tumors.^16^ In our study, the prognostic value of TMS was more pronounced in RCC than in LCC, supporting subsite-specific differences in its clinical utility.

Several limitations should be acknowledged. First, assessment of the individual TMS components is subject to methodological challenges. In the TSP assessment, distinguishing stromal tissue from smooth muscle fibers may be difficult.^9^ TB should be interpreted cautiously in specific histological subtypes, including medullary, mucinous, and signet-ring cell carcinomas, to avoid misclassifying structures that may resemble tumor buds.^10^ In addition, separating true buds from surrounding tissue can be challenging, particularly in areas with prominent inflammatory infiltrates.^10^ For KMG, H&E-based assessment does not differentiate between inflammatory cell types, and immunohistochemistry-based evaluation of inflammatory infiltrates may provide additional information.^17^ Interobserver variation is also a potential source of error, although TSP has shown high reproducibility,^9^ KMG demonstrates good agreement between observers,^8^ and tumor budding benefits from standardized recommendations established by the International Tumor Budding Consensus Conference.^10^ In the TMS algorithm used in this study, TB contributes to the TMS category only among tumors with low KMG (KMG 0-1), which should be considered when interpreting the relative contribution of the individual components. Second, the cohorts span 2000 to 2015, during which treatment paradigms may have changed and potentially confounded survival analyses. Third, all markers were assessed from a representative H&E slide showing the deepest tumor invasion. This approach was intended to capture the most aggressive component of the tumor but may not fully account for intratumoral heterogeneity. Finally, 2 independent evaluators were not used for all cases, although unclear cases were reviewed jointly under the supervision of an experienced pathologist (J.P.V.). This was considered acceptable because TSP and KMG have shown good interobserver reproducibility, and standardized assessment recommendations are available for tumor budding.^8–10^

A major strength of this study is its relatively large sample size, comprising 2 cohorts from 3 hospitals. To ensure sufficient statistical power, the Northern Finland validation cohorts from Oulu University Hospital and Lapland Central Hospital were combined. Complete follow-up data from the national registry, which records all deaths in Finland, further enhances the reliability of the survival analyses. Another strength is that TMS is derived directly from routine H&E-stained sections, which may facilitate its implementation in standard pathology workflows. This parallels tumor budding, which is incorporated into risk stratification in stage II colon cancer. In the ASCO guideline, high tumor budding, defined as ≥10 buds, is listed among high-risk features for which adjuvant chemotherapy may be offered.^18^ In this context, higher TMS categories could be evaluated as an additional H&E-based risk marker to support adjuvant treatment consideration, particularly in right-sided colon cancer, where its prognostic association appears strongest.

In conclusion, higher TMS categories were associated with poor prognosis in colon cancer. The prognostic impact of TMS was more pronounced in RCC than in LCC. These findings support TMS as a practical histopathological marker with potential to improve prognostic stratification in colon cancer patients.

## Supplementary Material
