# Supplementary material for: The Tumor Microenvironment Score and Its Subsite-Specific Relevance in Colon Cancer
Source: Am J Surg Pathol. 2026 Aug 25;50(11):1333–41. doi: 10.1097/PAS.0000000000002599 (PMC13657390; doi:10.1097/PAS.0000000000002599)
Supplement: Supplementary file 1 [file pas-50-1333-s001.docx]

# SUPPLEMENTAL DIGITAL CONTENT 1

Table S1: Multivariable analysis of 5-year disease-specific survival according to the tumor microenvironment score (TMS) in right-sided colon cancer (RCC) and left-sided colon cancer (LCC) in the discovery and validation cohort.

|  | **Multivariable analysis (RCC)** | | **Multivariable analysis (LCC)** | |
| --- | --- | --- | --- | --- |
|  | HR | p-value | HR | p-value |
| **Discovery cohort** | | | | |
| **TMS (Ref TMS0)** |  | 0.007 |  | 0.004 |
| - **TMS1** | 2.21 (1.34–3.66) | 0.002 | 1.65 (0.86–3.18) | 0.13 |
| - **TMS2** | 2.14 (1.28–3.59) | 0.004 | 2.77 (1.49–5.15) | 0.001 |
| - **TMS3** | 2.47 (1.36–4.46) | 0.003 | 3.08 (1.46–6.45) | 0.003 |
| **Age** | 1.02 (1.01–1.04) | 0.015 | 1.01 (0.99–1.04) | 0.29 |
| **Sex, female** | 0.78 (0.56–1.09) | 0.14 | 0.83 (0.55–1.24) | 0.35 |
| **Stage (Ref 1)** |  | <0.001 |  | <0.001 |
| - **2** | 2.02 (0.70–5.85) | 0.19 | 1.51 (0.43–5.27) | 0.52 |
| - **3** | 5.05 (1.78–14.28) | 0.002 | 3.05 (0.89–10.45) | 0.077 |
| - **4** | 21.19 (7.42–60.53) | <0.001 | 17.40 (4.99–50.65) | <0.001 |
| ***BRAF*, mutated** | 1.67 (1.05–2.67) | 0.03 | 2.11 (0.90–4.95) | 0.087 |
| **MMR, deficient** | 0.63 (0.37–1.11) | 0.11 | 1.07 (0.39–2.94) | 0.89 |
| **Lymphatic or vascular invasion, yes** | 1.88 (1.30-2.71) | <0.001 | 1.23 (0.78-1.91) | 0.37 |
| **ASA (Ref 1)** |  | 0.003 |  | <0.001 |
| - **2** | 0.61 (0.23–1.59) | 0.31 | 1.26 (0.38–4.24) | 0.71 |
| - **3** | 0.89 (0.34–2.36) | 0.81 | 1.49 (0.42–5.28) | 0.54 |
| - **4** | 1.59 (0.57–4.44) | 0.38 | 5.27 (1.45–19.19) | 0.012 |
| **Validation cohort** | | | | |
| **TMS (Ref TMS0)** |  | <0.001 |  | 0.146 |
| - **TMS1** | 1.91 (1.06–3.45) | 0.031 | 1.53 (0.74–3.17) | 0.25 |
| - **TMS2** | 1.93 (1.06–3.51) | 0.030 | 2.16 (1.10–4.26) | 0.026 |
| - **TMS3** | 4.35 (2.32–8.14) | <0.001 | 2.21 (0.81–6.03) | 0.12 |
| **Age** | 1.03 (1.00–1.05) | 0.026 | 1.02 (0.99–1.05) | 0.089 |
| **Sex, female** | 0.83 (0.56–1.24) | 0.36 | 1.15 (0.71–1.84) | 0.58 |
| **Stage (Ref 1)** |  | <0.001 |  | <0.001 |
| - **2** | 2.13 (0.79–5.79) | 0.14 | 0.32 (0.092–1.11) | 0.073 |
| - **3** | 3.80 (1.44–10.05) | 0.007 | 2.25 (0.91–5.57) | 0.079 |
| - **4** | 21.43 (7.89–58.30) | <0.001 | 14.63 (6.15–34.81) | <0.001 |
| ***BRAF*, mutated** | 1.48 (0.91–2.39) | 0.11 | 3.55 (1.15–10.92) | 0.027 |
| **MMR, deficient** | 0.78 (0.43–1.40) | 0.40 | 0.63 (0.12–3.42) | 0.60 |
| **Lymphatic or vascular invasion, yes** | 1.43 (0.95-2.17) | 0.086 | 1.48 (0.90–2.44) | 0.12 |
| **ASA (Ref 1)** |  | 0.037 |  | 0.31 |
| - **2** | 0.99 (0.37–2.66) | 0.98 | 1.27 (0.16–10.23) | 0.83 |
| - **3** | 1.05 (0.38–2.90) | 0.93 | 1.92 (0.24–15.60) | 0.54 |
| - **4** | 2.43 (0.78–7.60) | 0.13 | 2.66 (0.30–23.93) | 0.38 |

Abbreviations: ASA = American Society of Anesthesiologists Physical Status Classification System, KMG = The Klintrup-Mäkinen grade, LCC = Left-sided colon cancer, MMR =Mismatch-repair, N.A. =Not available, RCC = Right-sided colon cancer, TSP = Tumor stroma percentage
