# Supplementary material for: The Tumor Microenvironment Score and Its Subsite-Specific Relevance in Colon Cancer
Source: Am J Surg Pathol. 2026 Aug 25;50(11):1333–41. doi: 10.1097/PAS.0000000000002599 (PMC13657390; doi:10.1097/PAS.0000000000002599)
Supplement: Supplementary file 2 [file pas-50-1333-s002.docx]

**SUPPLEMENTAL DIGITAL CONTENT 1**

Table S2: Multivariable analysis of 5-year disease-specific survival according to the Glasgow microenvironment score (GMS) in right-sided colon cancer (RCC) and left-sided colon cancer (LCC) in the discovery and validation cohort.

|  | **Multivariable analysis (RCC)** | | **Multivariable analysis (LCC)** | |
| --- | --- | --- | --- | --- |
|  | HR | p-value | HR | p-value |
| **Discovery cohort** | | | | |
| **GMS (Ref GMS0)** |  | 0.002 |  | 0.003 |
| - **GMS1** | 2.35 (1.45–3.82) | <0.001 | 1.78 (0.95–3.35) | 0.073 |
| - **GMS2** | 2.07 (1.25–3.43) | 0.005 | 2.80 (1.52–5.14) | <0.001 |
| **Age** | 1.02 (1.01–1.04) | 0.013 | 1.01 (0.99–1.04) | 0.31 |
| **Sex, female** | 0.79 (0.56–1.10) | 0.16 | 0.84 (0.56–1.25) | 0.39 |
| **Stage (Ref 1)** |  | <0.001 |  | <0.001 |
| - **2** | 2.05 (0.71–5.93) | 0.19 | 1.53 (0.44–5.34) | 0.50 |
| - **3** | 5.17 (1.83–14.63) | 0.002 | 3.08 (0.90–10.54) | 0.073 |
| - **4** | 21.90 (7.67–62.52) | <0.001 | 18.42 (5.32–63.82) | <0.001 |
| ***BRAF*, mutated** | 1.70 (1.06–2.70) | <0.026 | 2.07 (0.87–4.92) | 0.099 |
| **MMR, deficient** | 0.60 (0.35–1.06) | 0.078 | 1.16 (0.42–3.19) | 0.78 |
| **Lymphatic or vascular invasion, yes** | 1.97 (1.38-2.83) | <0.001 | 1.23 (0.79-1.91) | 0.36 |
| **ASA (Ref 1)** |  | 0.002 |  | <0.001 |
| - **2** | 0.60 (0.23–1.56) | 0.30 | 1.27 (0.38–4.26) | 0.70 |
| - **3** | 0.88 (0.33–2.33) | 0.80 | 1.54 (0.44–5.40) | 0.50 |
| - **4** | 1.58 (0.56–4.41) | 0.38 | 5.33 (1.46–19.34) | 0.011 |
| **Validation cohort** | | | | |
| **GMS (Ref GMS0)** |  | 0.003 |  | 0.062 |
| - **GMS1** | 1.98 (1.11–3.53) | 0.021 | 1.53 (0.75–3.14) | 0.25 |
| - **GMS2** | 2.59 (1.51–4.45) | <0.001 | 2.18 (1.13–4.24) | 0.021 |
| **Age** | 1.02 (0.99–1.04) | 0.089 | 1.02 (0.99–1.05) | 0.090 |
| **Sex, female** | 0.85 (0.57–1.26) | 0.42 | 1.13 (0.70–1.82) | 0.63 |
| **Stage (Ref 1)** |  | <0.001 |  | <0.001 |
| - **2** | 2.17 (0.80–5.88) | 0.13 | 0.32 (0.09–1.12) | 0.074 |
| - **3** | 4.34 (1.66–11.36) | 0.003 | 2.27 (0.92–5.57) | 0.075 |
| - **4** | 22.84 (8.48–61.54) | <0.001 | 14.76 (6.23–34.93) | <0.001 |
| ***BRAF*, mutated** | 1.62 (0.99–2.62) | 0.051 | 3.54 (1.15–10.85) | 0.027 |
| **MMR, deficient** | 0.79 (0.43–1.45) | 0.44 | 0.64 (0.12–3.47) | 0.608 |
| **Lymphatic or vascular invasion, yes** | 1.39 (0.92-2.10) | 0.12 | 1.47 (0.90–2.42) | 0.12 |
| **ASA (Ref 1)** |  | 0.05 |  | 0.26 |
| - **2** | 0.82 (0.31–2.19) | 0.69 | 1.27 (0.16–10.32) | 0.82 |
| - **3** | 0.86 (0.32–2.30) | 0.76 | 1.93 (0.24–15.68) | 0.54 |
| - **4** | 1.91 (0.64–5.77) | 0.25 | 2.76 (0.31–24.74) | 0.37 |

Abbreviations: ASA = American Society of Anesthesiologists Physical Status Classification System, KMG = The Klintrup-Mäkinen grade, LCC = Left-sided colon cancer, MMR =Mismatch-repair, N.A. =Not available, RCC = Right-sided colon cancer, TSP = Tumor stroma percentage
