# Supplementary material for: The Tumor Microenvironment Score and Its Subsite-Specific Relevance in Colon Cancer
Source: Am J Surg Pathol. 2026 Aug 25;50(11):1333–41. doi: 10.1097/PAS.0000000000002599 (PMC13657390; doi:10.1097/PAS.0000000000002599)
Supplement: Supplementary file 3 [file pas-50-1333-s003.docx]

**SUPPLEMENTAL DIGITAL CONTENT 1**

Table S3: Univariable and multivariable analyses of TMS in subgroups of pMMR- and dMMR patients in both discovery and validation cohorts. Multivariable analysis was adjusted for BRAF status (wild-type/mutated), stage (I–II vs III–IV), age (continuous), sex (male/female), ASA class (1–2 vs 3–4), and lymphatic and/or vascular invasion (no/yes).

|  | **Univariable analysis (pMMR)** | | **Multivariable analysis (pMMR)** | | **Univariable analysis**  **(dMMR)** | | **Multivariable analysis**  **(dMMR)** | |
| --- | --- | --- | --- | --- | --- | --- | --- | --- |
|  | HR | p-value | HR | p-value | HR | p-value | HR | p-value |
| **Discovery cohort (Right-sided colon cancer)** | | | | | | | | |
| **TMS (Ref TMS0)** |  | <0.001 |  | 0.005 |  | 0.073 |  | 0.47 |
| - **TMS1** | 3.92  (2.15–7.12) | <0.001 | 2.78  (1.51–5.10) | 0.001 | 2.63  (1.10–6.33) | 0.031 | 2.10  (0.83–5.32) | 0.12 |
| - **TMS2** | 5.21  (2.93–9.36) | <0.001 | 2.10  (1.13–3.92) | 0.019 | 3.15  (1.20–8.29) | 0.02 | 1.87  (0.66–5.31) | 0.24 |
| - **TMS3** | 9.21  (4.97-17.07) | <0.001 | 2.94  (1.49–5.82) | 0.002 | 2.09  (0.27–16.30) | 0.48 | 1.69  (0.20–14.26) | 0.63 |
| **Validation cohort (Right-sided colon cancer)** | | | | | | | | |
| **TMS (Ref TMS0)** |  | <0.001 |  | <0.001 |  | 0.027 |  | 0.57 |
| - **TMS1** | 2.83  (1.46–5.51) | 0.002 | 2.81  (1.41–5.60) | 0.003 | 1.72  (0.53–5.59) | 0.37 | 1.38  (0.42–4.61) | 0.60 |
| - **TMS2** | 3.26  (1.72–6.17) | <0.001 | 3.26  (1.68–6.34) | <0.001 | 0.66  (0.083–5.17) | 0.69 | 0.78  (0.094–6.45) | 0.82 |
| - **TMS3** | 8.06  (4.17–15.56) | <0.001 | 5.21  (2.58–10.53) | <0.001 | 5.75  (1.76–18.83) | 0.004 | 2.54  (0.66–9.79) | 0.18 |
| **Discovery cohort (Left-sided colon cancer)** | | | | | | | | |
| **TMS (Ref TMS0)** |  | <0.001 |  | <0.001 | N.A* |  | N.A* |  |
| - **TMS1** | 2.16  (1.15–4.06) | 0.017 | 1.59  (0.84–3.02) | 0.16 |  |  |  |  |
| - **TMS2** | 5.30  (2.98–9.44) | <0.001 | 3.51  (1.94–6.37) | <0.001 |  |  |  |  |
| - **TMS3** | 8.54  (4.35–16.77) | <0.001 | 5.13  (2.54–10.37) | <0.001 |  |  |  |  |
| **Validation cohort (Left-sided colon cancer)** | | | | | | | | |
| **TMS (Ref TMS0)** |  | <0.001 |  | 0.007 | N.A* |  | N.A* |  |
| - **TMS1** | 1.78  (0.88–3.59) | 0.11 | 2.01  (0.98–4.11) | 0.055 |  |  |  |  |
| - **TMS2** | 4.88  (2.59–9.19) | <0.001 | 3.14  (1.64–6.00) | <0.001 |  |  |  |  |
| - **TMS3** | 3.00  (1.14–7.90) | 0.026 | 1.89  (0.71–5.04) | 0.20 |  |  |  |  |

Abbreviations: dMMR = Deficient mismatch-repair, N.A = Not available, pMMR = Proficient mismatch-repair

*Cox regression analysis was not feasible due to small subgroup sizes
