# Supplementary material for: The Tumor Microenvironment Score and Its Subsite-Specific Relevance in Colon Cancer
Source: Am J Surg Pathol. 2026 Aug 25;50(11):1333–41. doi: 10.1097/PAS.0000000000002599 (PMC13657390; doi:10.1097/PAS.0000000000002599)
Supplement: Supplementary file 4 [file pas-50-1333-s004.docx]

**SUPPLEMENTAL DIGITAL CONTENT 1**

Table S4: Univariable and multivariable analyses of TMS in subgroups of stage II and III patients in both discovery and validation cohorts. No statistically significant associations were observed for stages I and IV. Multivariable analysis was adjusted for BRAF status (wild-type/mutated), Mismatch-repair status (proficient/deficient), age (continuous), sex (male/female), ASA class (1–2 vs 3–4), and lymphatic and/or vascular invasion (no/yes).

|  | **Univariable analysis (Stage II)** | | **Multivariable analysis (Stage II)** | | **Univariable analysis**  **(Stage III)** | | **Multivariable analysis**  **(Stage III)** | |
| --- | --- | --- | --- | --- | --- | --- | --- | --- |
|  | HR | p-value | HR | p-value | HR | p-value | HR | p-value |
| **Discovery cohort (Right-sided colon cancer)** | | | | | | | | |
| **TMS (Ref TMS0)** |  | 0.015 |  | 0.010 |  | 0.010 |  | 0.090 |
| - **TMS1** | 2.12  (0.77–5.85) | 0.15 | 2.07  (0.72–5.96) | 0.177 | 1.98  (0.88–4.46) | 0.099 | 1.57  (0.68–3.64) | 0.29 |
| - **TMS2** | 4.84  (1.86–12.57) | 0.001 | 5.20  (1.97–13.76) | <0.001 | 2.00  (0.95–4.20) | 0.68 | 1.32  (0.59–2.93) | 0.50 |
| - **TMS3** | 2.23  (0.28–17.84) | 0.45 | 1.38  (0.16–12.15) | 0.770 | 3.98  (1.76–8.96) | <0.001 | 2.83  (1.16–6.93) | 0.023 |
| **Validation cohort (Right-sided colon cancer)** | | | | | | | | |
| **TMS (Ref TMS0)** |  | 0.024 |  | 0.043 |  | 0.031 |  | 0.038 |
| - **TMS1** | 2.29  (0.61–8.52) | 0.22 | 1.73  (0.45–6.73) | 0.42 | 2.50  (0.99–6.30) | 0.052 | 2.30  (0.90–5.84) | 0.081 |
| - **TMS2** | 3.92  (0.93–10.91) | 0.064 | 3.03  (0.83–11.03) | 0.093 | 2.04  (0.79–5.28) | 0.144 | 1.82  (0.66–5.02) | 0.25 |
| - **TMS3** | 10.09  (2.26–45.09) | 0.002 | 12.44  (2.07–74.86) | 0.006 | 3.77  (1.56–9.15) | 0.003 | 3.90  (1.54–9.88) | 0.004 |
| **Discovery cohort (Left-sided colon cancer)** | | | | | | | | |
| **TMS (Ref TMS0)** |  | 0.60 |  | 0.82 |  | 0.45 |  | 0.32 |
| - **TMS1** | 0.85  (0.28–2.59) | 0.77 | 0.95  (0.30–3.02) | 0.93 | 0.96  (0.26–3.58) | 0.96 | 0.92  (0.24–3.58) | 0.90 |
| - **TMS2** | 1.01  (0.30–3.35) | 0.99 | 1.24  (0.36–4.31) | 0.73 | 1.70  (0.51–5.64) | 0.39 | 1.86  (0.53–6.49) | 0.33 |
| - **TMS3** | 2.57  (0.55–12.10) | 0.23 | 2.06  (0.37–11.58) | 0.41 | 1.66  (0.46–6.00) | 0.44 | 1.31  (0.34–4.96) | 0.70 |
| **Validation cohort (Left-sided colon cancer)** | | | | | | | | |
| **TMS (Ref TMS0)** | N.A* |  | N.A* |  |  | <0.001 |  | <0.001 |
| - **TMS1** |  |  |  |  | 1.96  (0.28–13.94) | 0.50 | 2.44  (0.32–18.52) | 0.39 |
| - **TMS2** |  |  |  |  | 14.36  (3.26–63.27) | <0.001 | 21.75  (4.3–110.00) | <0.001 |
| - **TMS3** |  |  |  |  | 2.24  (0.20–24.70) | 0.51 | 2.91  (0.24–34.81) | 0.40 |

Abbreviations: N.A = Not available

*Cox regression analysis was not feasible due to small subgroup sizes
